# The IFN-Ɣ + 874 A/T polymorphism is associated with malignant breast cancer in a population from the southwest of Iran

**DOI:** 10.1186/s13104-021-05543-6

**Published:** 2021-04-20

**Authors:** Hadi Rezaeean, Gholam Abbas Kaydani, Najmaldin Saki, Sasan Razmjoo, Maryam Labibzadeh, Hamid Yaghooti

**Affiliations:** 1 High Institute for Education and Research in Transfusion Medicine, Tehran, Iran; 2 Thalassemia and Hemoglobinopathy Research Center, Ahvaz Jundishapur University of Medical Sciences, Ahvaz, Iran; 3 Department of Clinical Oncology, Ahvaz Jundishapur University of Medical Sciences, Ahvaz, Iran; 4 Department of Medical Laboratory Sciences, School of Allied Medical Sciences, Ahvaz Jundishapur University of Medical Sciences, Ahvaz, Iran; 5 Hyperlipidemia Research Center, Ahvaz Jundishapur University of Medical Sciences, Ahvaz, Iran

**Keywords:** Interferon-Ɣ, Polymorphism, Breast cancer, Malignant

## Abstract

**Objective:**

Breast cancer (BC) is one of the most common diseases in women globally, with an increasing number of deaths associated with it. Recently the role of polymorphisms in the genes encoding cytokines and immune cells has been demonstrated. This study aimed to evaluate the association of IFN-Ɣ + 874 A/T polymorphism with BC clinical symptoms.

**Results:**

The study included 88 women with BC and 88 healthy women who had no history of cancer and were matched for age and sex. Allele-specific oligonucleotide-polymerase chain reaction technique was used to investigate the IFN-Ɣ polymorphism. Clinical data were obtained from the patients’ records. Our results showed that the frequencies of genotypes in the BC patients were not significantly different from the control subjects. However, in the patients, the AT genotype was associated with the risk of malignant BC. The age at BC diagnosis was not different in patients with AA and AT genotypes; however, it was significantly earlier in HER2 negative subjects (p = 0.002). Given the higher frequency of AT in malignant BC patients, our results confirm the association of the IFN-Ɣ polymorphism with the disease’s progression to a malignant state.

**Supplementary Information:**

The online version contains supplementary material available at 10.1186/s13104-021-05543-6.

## Introduction

Breast cancer (BC) is the most common cancer in women accounting for nearly 14% of all cancer-related deaths [1]. The immune system is one of the factors that have a dual role in cancer. On the one hand, this system prevents angiogenesis and proliferation of cancer cells, and on the other hand, it promotes the formation and metastasis of cancer cells to different parts of the body [2, 3]. Interferons (IFNs) are a family of cytokines involved in regulating immune system responses against viral infection and cancer cells [4]. It is produced predominantly by natural killer (NK) and natural killer T (NKT) cells as part of the innate immune response and by CD4 Th1 and CD8 cytotoxic T lymphocytes [5].

The IFN-Ɣ coding gene is located on chromosome 12 inthe 12q24 region, consisting of 4 exons and three introns [6]. Recently a single nucleotide polymorphism (SNP) has been identified at position + 874 (IFN-Ɣ + 874 A/T, rs2430561) at intron 1 of the IFN-Ɣ coding gene. The + 874 region is the binding site of the nuclear factor kappa light chain enhancer of activated B cells (NF-kB) involved in the production of IFN-Ɣ. The A to T transversion in the + 874 region causes AA, AT, and TT genotypes to decrease, average, and increase IFN-Ɣ production, respectively [7]. Given that IFN-Ɣ is involved in the expression of immune system responses against cancer cells, recent studies have shown that the rs2430561 polymorphism is involved in the BC’s pathogenesis. It has been reported that the frequency of the IFN-Ɣ T/T genotype was higher in BC patients than in the control group, and the T/T genotype increased the risk of BC [8]. However, other studies showed no relationship between IFN-Ɣ + 874 A/T polymorphism and the BC’s risk [9]. The relationship between IFN-Ɣ polymorphism and BC patients’ pathophysiological symptoms has not been evaluated so far in our population. Suppose the association of this genetic factor with the incidence and clinical condition of BC be demonstrated in a population. In that case, it can be used as a prognostic marker to evaluate the disease. Therefore, in this study, we investigated the prevalence of IFN-Ɣ + 874 A/T polymorphism in cancer and healthy individuals and its association with the patients’ pathologic markers and clinical status.

## Main text

### Materials and methods

#### Study population

Eighty-eight women with BC and Eighty-eight healthy women who had no family history of cancer and other malignancies were included in the study. Patients were either new cases or treated, referred to the Golestan Hospital in Ahvaz, southwest of Iran, between 2014 and 2017. The mean age of the patients was 47.2 ± 9.2. The controls also had the same age and sex as the patients. Before selecting control subjects, they were asked about their previous history and that of their family, and individuals with no family history of cancer and no present disease were selected. These people were selected from the clients who were referred to the laboratory for a checkup. The selection of BC patients was based on their records and an oncologist’s diagnosis, with no concomitant disease. This study was approved by the ethics committee of Ahvaz Jundishapur University of Medical Sciences (IR.AJUMS.REC.1396.717), and informed consent was obtained from each subject participating in the study.

#### DNA extraction

Approximately 2 mL of peripheral blood was collected from all subjects in acetic acid-containing anticoagulant tubes. DNA was extracted from blood samples using Yektatajhiz Azma kit (Iran) according to the manufacturer's protocol. The extracted DNAs were stored at − 20 °C until analysis.

#### DNA analysis

IFN-Ɣ + 874A/T polymorphism was analyzed by allele-specific oligonucleotide polymerase chain reaction (ASO-PCR). The sequence of primers used is shown in Table 1 [8]. Two separate microtubes were used for each sample, all of which were identical for each PCR reaction with reverse primer, but the forward primers were different and specific to the A and T alleles in each tube. The reaction solution volume was 25 µL containing 10 µL distilled water (DW), 10 μmol PCR 2× Master mix, 200 ng DNA, 20 pmol forward, and 20 pmol reverse primers. Conditions for performing PCR cycles for 35 cycles were as follow 95 °C for 5 min, 95 °C for 45 s, 55 °C for 45 s, 72 °C for 1 min with final extension for 3 min at 72 °C.

**Table 1:** Primers used for the detection of IFN-Ɣ + 874 A/T polymorphism

Primer	Sequence	Product length
Forward	5′-ttcttacaacacaaaatcaaatca-3'	262 bp
	5′-ttcttacaacacaaaatcaaatct-3'	
Reverse	5′-tcaacaaagctgatactcca-3'	

The PCR products were then characterized by 2% agarose gel electrophoresis. The bands associated with the AA and AT genotypes were both 262 bp in length.

#### Statistical analysis

To describe the data, we used frequencies and percentages. A Chi-square test was used to compare the allele and genotype frequencies. Odds Ratio (OR) was calculated using the Mantel–Haenszel test to evaluate the association between AA and AT genotypes and BC and its clinical parameters. The Mann*–*Whitney U test was applied for comparison of the quantitative variables*.* All analyzes were performed using SPSS version 22. *P* < 0.05 was considered to be statistically significant.

### Results

#### Prevalence of the IFN-Ɣ + 874 A/T genotypes in the patients and control subjects

Frequencies of IFN-Ɣ + 874 A/T genotypes and alleles in our population are shown in Table 2. It was found that the prevalent genotype was AT in both groups, whereas no subject with TT genotype was detected. We also found no statistically significant difference between the frequency of genotypes in the control group and patients. The OR for AA genotype versus AT genotype was 0.92 (95% CI 0.4–2.1, p-value = 0.8) for BC.

**Table 2:** Genotype and allele frequencies in patients and control subjects

	AA	AT	TT	A	T	*p*-value
Breast cancer	13 (14.8%)	75 (85.2%)	0 (0%)	101 (57.4%)	75 (42.6%)	0.8
Controls	14 (15.9%)	74 (84.1%)	0 (0%)	102 (58%)	74 (42%)	0.9
OR (95% CI)	1.0-ref	0.9(0.4–2.1)		1.0-ref	0.98 (0.7–1.4)	

Data are expressed as number (%)

#### Association between IFN-Ɣ + 874 A/T variants and clinical parameters

We extracted data from patients’ records to evaluate the relationship between patients’ polymorphism and clinical symptoms. We examined the association of each of the recorded clinical parameters with the polymorphism genotypes. The results showed that AT genotype frequency in both benign and malignant cancer patients was higher than that of AA genotype. However, the percentage of patients with the AT genotype was higher in malignant patients than the benign patients. The OR for AT genotype versus the AA genotype to have a malignant phenotype was 5.3 (95% CI 1.1–26, p-value = 0.05). For other recorded parameters of the disease, including the occurrence of metastasis, right versus left affected breast, and HER2, ER, and PR status, the frequency of the AT genotype was higher than that of AA genotype. However, the IFN-Ɣ + 874 A/T AT genotype was not significantly associated with any of these clinical parameters (Table 3). The alleles of the polymorphism also did not show significant associations with the parameters. Similarly, the genotypes’ frequency was not different in patients with stages I to IV of BC based on a Chi-square analysis (Additional file 1: Table S1).

**Table 3:** Association of the the IFN-Ɣ + 874 A/T genotypes with clinical parameters of BC

	AA 1.0-ref	AT	OR for ATvsAA (95% CI)	*p*-value	A 1.0-ref	T	OR for TvsA (95% CI)	*p*-value
Malignancy	3 (6.5)	44 (93.5)	5.3 (1.1–26)	0.05	50 (53.2)	44 (46.8)	1.5 (0.7–3)	0.3
Benign	11 (27)	30 (73)			52 (62.5)	30 (37.5)		
Metastasis	10 (13.5)	64 (68.5)	1.7 (0.4–7.4)	0.4	84 (56.8)	64 (43.2)	1.2 (0.5–2.7)	0.7
No metastasis	3 (21.4)	11 (78.6)			17 (60.7)	11 (39.3)		
Right	7 (17.1)	34 (82.9)	0.7 (0.2–2.7)	0.6	48 (58.5)	34 (41.5)	1.1 (0.6–2)	0.8
Left	6 (13)	40 (87)			52 (56.5)	40 (43.5)		
HER 2 positive	6 (11.3)	47 (88.7)	2.3 (0.6–8.5)	0.2	59 (55.7)	47 (44.3)	0.8 (0.4–1.6)	0.5
HER 2 negative	5 (22.7)	17 (77.3)			27 (61.4)	17 (38.6)		
PR positive	8 (14.3)	48 (85.7)	1.1 (0.3–4.8)	0.87	64 (57.1)	48 (42.9)	0.97 (0.5–2)	0.9
PR negative	3 (15.8)	16 (84.2)			22 (57.9)	16 (42.1)		
ER positive	8 (13.3)	52 (86.7)	1.6 (0.4–7)	0.5	68 (56.7)	52 (43.3)	0.87 (0.4–1.97)	0.7
ER negative	3 (20)	12 (80)			18 (60)	12 (40)		

Data are expressed as number (%)

Also, we analyzed two other clinical parameters concerning the genotypes. The patients’ ages at the first diagnosis of BC and tumor size were recorded in the patients and were compared in the AA and AT genotypes. The age of diagnosis was 46.8 and 47.3 years in patients with AA and AT genotype, respectively (p = 0.6), and tumor size was 2.6 vs. 2.7 for these genotypes, respectively (p = 0.97). The differences were not statistically significant. However, it was notable that the age of diagnosis was significantly different concerning HER2. It was found that HER2 negative patients represent earlier BC diagnosis age than HER2 positive patients (42.4 vs. 48.9, p = 0.002).

### Discussion

BC is one of the most common neoplasms worldwide, with an increased incidence of women’s deaths over the past few years [10, 11]. Various therapies have been used to control the spread of BC so far. However, no effective treatment can completely eradicate BC cells in the body and prevent recurrence after initial treatment [12].

There is also a significant relationship between mutations in genes encoding cytokines and BC [13]. Research to date has identified many mutations in the genes encoding inflammatory cytokines. However, among these mutations, the IFN-γ gene expression (rs2430561) appears to play an essential role in the pathogenesis of BC and its response to treatment [14]. For this purpose, the study of Kamali-Sarvestani et al. conducted on women with BC and healthy women showed that TT genotype frequency was higher in female patients than in healthy women. However, no significant association was found between IFN-Ɣ + 874 A/T polymorphism and the incidence of BC in women [8]. Also, the study by Gonullu et al. was performed to evaluate the association of IFN-Ɣ, IL-6, IL-10, tumor growth factor-β (TGF-β) and Tumor necrosis factor-α (TNF-α) polymorphisms with the disease. Mutations in the IL-6 and IL-10 coding genes were found to play a role in BC progression, whereas mutations in the IFN-1, TGF-β, and TNF-α coding genes did not play a role in BC pathogenesis [9]. On the other hand, Karakus et al. showed that AT and AA genotypes resulted from IFN-Ɣ + 874 A/T polymorphism and TNF-b þ252 GG from TNF-b þ252 (A>G) in women with BC were higher than in normal women. It was found that these genotypes promote the progression of BC cells [15].

Our results showed that the frequency of AT genotype was higher in BC and control group compared to AA genotype. However, the genotypes were not associated with the disease (p = 0.8). No TT genotype was observed in the patients, and no significant relationship between the AA/AT genotypes and the disease incidence.

Parihar et al.’s study showed that HER2-positive BC cells stimulate the immune system and increase this cytokine production due to polymorphisms in the interferon-gamma gene concomitant with the production of interferon-gamma in BC HER2-positive patients with reduced disease progression [16].

In a study by Esther et al., the results of their studies showed that there was no relationship between gamma interferon polymorphism and stage, disease, and lymph node involvement [17]. A study by Hao et al. showed that IFN-γ gene polymorphism is associated with increased metastasis of cancer cells and increased proliferation. Also, it was shown that the incidence of polymorphism in stages III and IV was higher than in stages I and II, indicating an impairment of IFN-γ secretion due to polymorphism and disease progression [18].

In the present study, the data analysis results showed a significant relationship between the polymorphism genotype and the malignant phenotype. This finding is in accordance with the earlier studies that the IFN-Ɣ + 874 A/T polymorphism is associated with the disease’s progression to a malignant phenotype. However, the polymorphism was not correlated with the disease stages after classification of the patients to four clinical stages. An essential parameter regarding BC is the age of presentation and diagnosis of the disease. The diagnosis age was not different in the AA and AT genotypes of IFN-Ɣ + 874 A/T polymorphism. However, it was found that the HER2 status of patients is a significant factor in this regard.

The frequency of single nucleotide polymorphisms and their clinical correlations are different among populations. A consensus conclusion regarding their significance cannot be achieved due to the limited number of BC subjects and the cross-sectional nature of the studies. Hence, the individual studies are necessary to conduct in different populations to be analyzed through a meta-analysis study.

### Conclusion

Although there was no statistically significant relationship between the frequency of genotypes and BC, our results showed that patients with the IFN-Ɣ + 874 A/T AT genotype were at higher risk of progression to a malignant state. Therefore, having an AA genotype would be in favor of a better prognosis of the disease.

## Limitation

- Patients were not followed up for response to treatment.
- The prognostic role of IFN-Ɣ + 874 A/T polymorphism of patients was not evaluated.
- The cytokine production related to IFN-Ɣ + 874 A/T polymorphism was not evaluated.

## Supplementary Information

**Additional file 1: Table S1.** Frequency of the AA and AT genotypes in different stages of BC.

## Data Availability

The datasets used analyzed during the current study are available from the corresponding author on reasonable request.

## Abbreviations

BC: Breast cancer
INF: Interferon
SNP: Single nucleotide polymorphism
