# Supplementary material for: The IFN-Ɣ + 874 A/T polymorphism is associated with malignant breast cancer in a population from the southwest of Iran
Source: BMC Res Notes. 2021 Apr 20;14:147. doi: 10.1186/s13104-021-05543-6 (PMC8056653; doi:10.1186/s13104-021-05543-6)
Supplement: Supplementary file 1 — Additional file 1: Table S1. Frequency of the AA and AT genotypes in different stages of BC. [file 13104_2021_5543_MOESM1_ESM.docx]

Additional file 1: Table. S1. Frequency of the AA and AT genotypes in different stages of BC

| stage | AA | AT | P value |
| --- | --- | --- | --- |
| I | 0 | 6 (100) | 0.5 |
| II | 7 (13) | 47 (87) |  |
| III | 5 (31.3) | 11 (68.8) |  |
| IV | 1 (8.3) | 11 (91.7) |  |

Data are expressed as number (%)
